# Repurposing novel therapeutic candidate drugs for coronavirus disease-19 based on protein-protein interaction network analysis

**DOI:** 10.1186/s12896-021-00680-z

**Published:** 2021-03-12

**Authors:** Masoumeh Adhami, Balal Sadeghi, Ali Rezapour, Ali Akbar Haghdoost, Habib MotieGhader

**Affiliations:** 1 Pathology and Stem Cell Research Center, Kerman University of Medical Sciences, Kerman, Iran; 2 Food Hygiene and Public Health Department, Faculty of Veterinary Medicine, Shahid Bahonar University of Kerman, Kerman, Iran; 3 Department of Agriculture, Tabriz Branch, Islamic Azad University, Tabriz, Iran; 4 Modeling in Health Research Center, Institute for Futures Studies in Health, Kerman University of Medical Sciences, Kerman, Iran; 5 Department of Basic sciences, Biotechnology Research Center, Tabriz Branch, Islamic Azad University, Tabriz, Iran; 6 Department of Computer Engineering, Gowgan Educational Center, Tabriz Branch, Islamic Azad University, Tabriz, Iran

## Abstract

**Background:**

The coronavirus disease-19 (COVID-19) emerged in Wuhan, China and rapidly spread worldwide. Researchers are trying to find a way to treat this disease as soon as possible. The present study aimed to identify the genes involved in COVID-19 and find a new drug target therapy. Currently, there are no effective drugs targeting SARS-CoV-2, and meanwhile, drug discovery approaches are time-consuming and costly. To address this challenge, this study utilized a network-based drug repurposing strategy to rapidly identify potential drugs targeting SARS-CoV-2. To this end, seven potential drugs were proposed for COVID-19 treatment using protein-protein interaction (PPI) network analysis. First, 524 proteins in humans that have interaction with the SARS-CoV-2 virus were collected, and then the PPI network was reconstructed for these collected proteins. Next, the target miRNAs of the mentioned module genes were separately obtained from the miRWalk 2.0 database because of the important role of miRNAs in biological processes and were reported as an important clue for future analysis. Finally, the list of the drugs targeting module genes was obtained from the DGIDb database, and the drug-gene network was separately reconstructed for the obtained protein modules.

**Results:**

Based on the network analysis of the PPI network, seven clusters of proteins were specified as the complexes of proteins which are more associated with the SARS-CoV-2 virus. Moreover, seven therapeutic candidate drugs were identified to control gene regulation in COVID-19. *PACLITAXEL*, as the most potent therapeutic candidate drug and previously mentioned as a therapy for COVID-19, had four gene targets in two different modules. The other six candidate drugs, namely, BORTEZOMIB, CARBOPLATIN, CRIZOTINIB, CYTARABINE, DAUNORUBICIN, and VORINOSTAT, some of which were previously discovered to be efficient against COVID-19, had three gene targets in different modules. Eventually, CARBOPLATIN, CRIZOTINIB, and CYTARABINE drugs were found as novel potential drugs to be investigated as a therapy for COVID-19.

**Conclusions:**

Our computational strategy for predicting repurposable candidate drugs against COVID-19 provides efficacious and rapid results for therapeutic purposes. However, further experimental analysis and testing such as clinical applicability, toxicity, and experimental validations are required to reach a more accurate and improved treatment. Our proposed complexes of proteins and associated miRNAs, along with discovered candidate drugs might be a starting point for further analysis by other researchers in this urgency of the COVID-19 pandemic.

**Supplementary Information:**

The online version contains supplementary material available at 10.1186/s12896-021-00680-z.

## Introduction

A novel coronavirus (i.e., COVID-19) has led to the emergence of a major outbreak in the world. Severe acute respiratory syndrome coronavirus 2 (SARS-CoV-2) is the main cause of coronavirus disease which has turned to become an international concern worldwide [1–4]. According to the latest reports of the COVID-19 situation in the world, as of 15 November 2020, 53.7 million confirmed cases and 1.3 million deaths occurred worldwide [5]. SARS-CoV-2 is a positive-sense single-stranded RNA genome. Based on the latest genome assemblies of this virus, it contains 12 proteins, 11 genes, GC 38%, and the size of 29.9 KB (https://www.ncbi.nlm.nih.gov/genome), with a 5′-cap structure and a 3′-poly-A tail [6].

miRNAs are small molecules of non-coding RNA that inhibit the translation of mRNAs in prokaryotes and eukaryotes [7, 8]. Recent evidence has confirmed the role of pathologic processes in miRNAs, including inflammatory responses and viral infection. It has been demonstrated that miR-9, miR-98, miR-223, and miR-214 expressions in COVID-19-infected host cells should be changed, and consequently, leading to amendments in cytokines manufacturing [9].

A distinguishing proof of host factors, especially genes for contamination is basic to educate systems regarding COVID-19 pathogenesis, uncover varieties in having powerlessness, and recognize novel host-coordinated treatments, which may have viability against current and future pandemic coronaviruses [10]. Recent research has represented and validated anti-viral genes (e.g., CABIN1, HIRA, and ASF1A). These genes potentially provide protection from SARS-CoV-2 [11, 12]. A number of studies have focused on the immune system [12–15]. Melo et al. [16] detailed a moderate interferon (IFN) reaction to SARS-CoV-2 disease in essential cells and indicated that IFN can decrease SARS-CoV-2 replication in vitro. Moreover, Zhou et al. [17] and Xiong et al. [18] analyzed the inborn invulnerable reaction to SARS-CoV-2 contamination in the bronchoalveolar lavage liquid (BALF). These examinations detailed the noteworthy upregulation of a subset of interferon-invigorated qualities (ISGs) which are legitimately identified with an antiviral action (e.g., ISG15, IFIH1, MX1, OAS1–3, and IFITMs). Prasad et al. reported that several genes of the host (e.g., OAS1–3, IRF7, IRF9, STAT1, and IFIH1) are exceptionally communicated and profoundly related to reactions to viral contaminations [19]. Likewise, Cava et al. found that nine genes (i.e., LRRK2, ACSL5, HSD17B4, EPHX1, MCCC2, GSTA4, ACACA, HGD, and ROS1) were positively correlated with angiotensin-converting enzyme 2 while one gene (CRIP2) was negatively correlated with this enzyme [20].

miRNA-based therapy could be proposed for SARS-CoV-2 treatment through viral genome suppression [21]. Antibodies or anther antiviral medications are not yet accessible for COVID-1 9 contamination medicines [22]. In a short time after this pandemic infection, many scientists seek to identify the involved host genes and proteins in diseases to find a new therapy [17, 23].

Concerning the current speed of SARS-CoV-2 spread in the world, the drug repurposing strategy is a more immediate and effective method of drug discovery [24, 25]. Recently, multiple studies have utilized network-based and computational approaches for repurposing candidate drugs for COVID-19 [26, 27]. Network-based approaches have demonstrated their effectiveness in the identification of repurposable drugs for various human diseases during the last decade [28, 29].

Considering the above-mentioned explanations, the present study aimed to identify the genes involved in COVID-19 and find a new drug target therapy. Currently, there are no effective drugs targeting SARS-CoV-2 and drug discovery approaches are considered costly and time-consuming. In this regard, this study applied a network-based drug repurposing strategy in order to rapidly identify potential drugs targeting SARS-CoV-2. For this purpose, we used protein-protein interaction (PPI) network and computational tools with resources for genomics and proteomics. Appropriate drugs might be useful for understanding viral disease mechanisms and designing and developing anti-viral agents.

## Material and methods

### Dataset and preprocessing

**Fig. 1:**
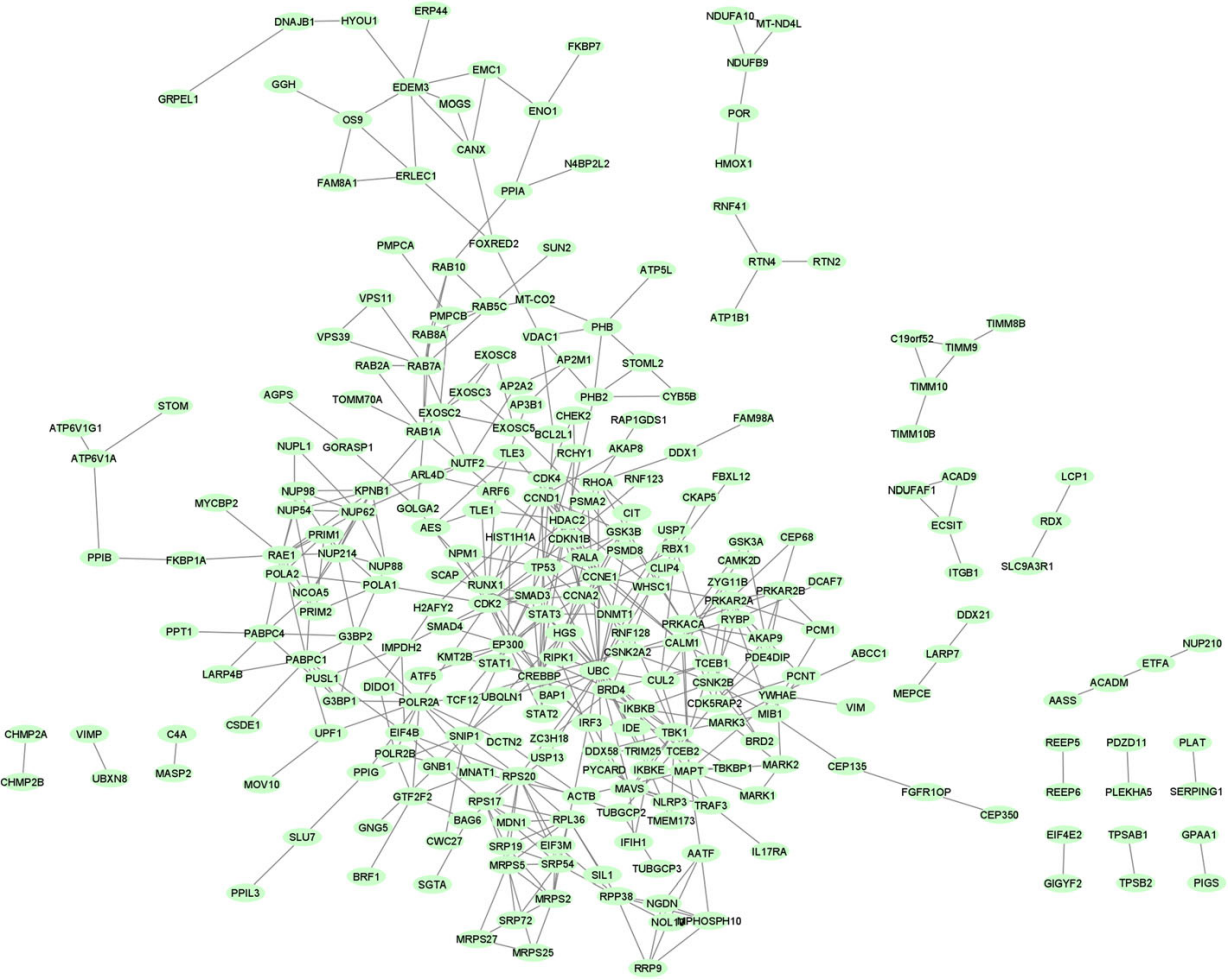
Global PPI network based on Gathered 524 Proteins. *Note*. PPI: Protein-protein interaction. Every node corresponds to a protein, and edges show experimentally validated physical interactions

In this work, coronavirus interaction data release was downloaded from the BioGRID database [30] containing the most up-to-date version of genes interacted in the COVID-19. This dataset contained 928 entries (https://downloads.thebiogrid.org/BioGRID/Latest-Release), and 524 genes remained after preprocessing. For preprocessing, all data were checked out and the duplicate gene symbols were eliminated, and then the missing gene symbols were extracted from the NCBI database using the Entrez ID and imported into the data. Next, the PPI network was extracted for our gene dataset (Fig. 1) by applying the STRING database, version 11.0 [31]. To this end, our search was limited to those experimentally validated interactors which had direct interaction with our gene list. Afterward, the PPI network was plotted using Cytoscape 3.6.0 [32].

### Clustering and network analysis

**Fig. 2:**
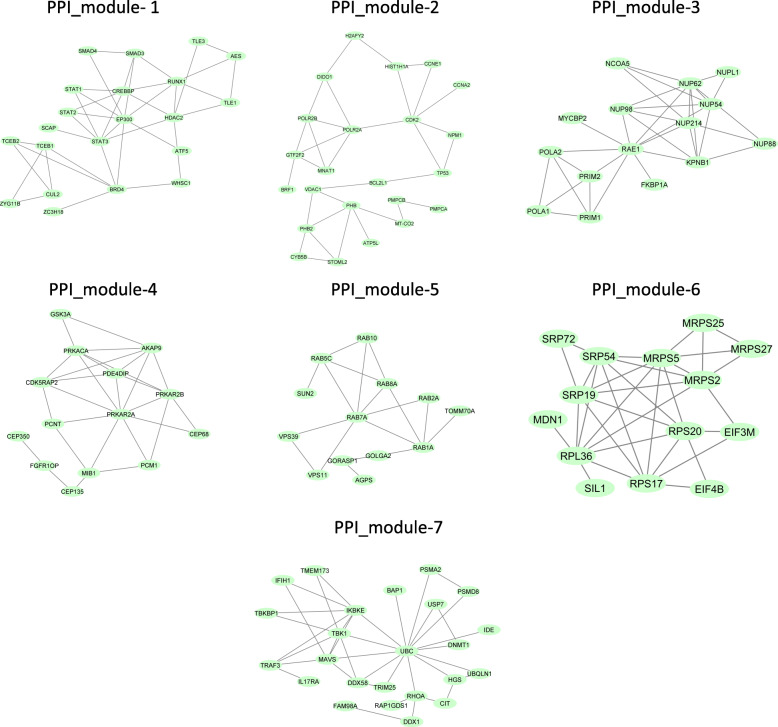
Obtained PPI modules from PPI networks. *Note*. Protein-protein interaction. The green circles represent each module genes

For the next analysis of this biological network, ClusterViz [33] was utilized for clustering the highly physically interconnected modules of proteins (protein complexes). The cluster analysis of biological networks (e.g., PPI or gene networks) is one of the most common strategies for detecting protein complexes or functional modules. Clustervize is a user-friendly and platform-independent plugin for cytoscape which facilitates the operation of the user [33]. ClusterViz uses different algorithms to perform clustering analysis. The fast agglomerate edge clustering (FAG-EC) algorithm was selected based on the aim of this study. FAG-EC is a fast agglomerative hierarchical clustering algorithm that functions based on edge clustering coefficients. First, this algorithm calculates the edge clustering coefficient for each edge in the network. Then, edges are sorted in a non-increasing order according to clustering coefficients. The complexes of proteins are detected according to the bottom-up condensing the hierarchical clustering algorithm. Due to the low complexity and fast computational power, the FAG-EC algorithm is effective for analyzing large protein networks [34]. The selected parameters of the algorithm included DefinitionWay: Weak, In/OutThreshold: 1.0, Overlapped: true, CliqueSizeThreshold: 3, and OutputThreshold: 10. Totally, seven different PPI clusters (i.e., PPI complexes) were discovered based on the analysis. Figure 2 shows these seven PPI modules, and the list of genes for every cluster (module) is provided in supplementary file S2.

### mRNA-miRNA bipartite sub-network reconstruction

In the next step, the experimentally validated miRNA targets of genes were searched in the miRWalk 2.0 database in order to reconstruct the bipartite of mRNA-miRNA sub-network for each PPI module [35]. Eventually, seven bipartite mRNA-miRNA sub-networks were obtained after removing duplicate connections. In these sub-networks, each node demonstrates the genes or their miRNA targets, and each edge shows the connection between the nodes. Moreover, the sub-networks were plotted and analyzed using Cytoscape 3.6.0 [32]. The degree of each node represents the number of connections of that node with other nodes of the network. A node with a higher degree has a more important and remarkable role in the network. In general, 10 miRNAs with the highest degree for each sub-network were determined in this phase of our analysis. The details of the reconstructed bipartite mRNA-miRNA sub-networks, along with network information are reported in supplementary file S2.

### Functional annotation and pathway enrichment analysis of the identified clusters of genes in SARS-CoV-2 infection

In the next phase, the functional enrichment analysis was performed for each sub-network in order to identify the biological mechanisms of important genes and miRNAs. The applied tools and databases were the gene ontology (GO) tool of DAVID^1^ [36] for functional enrichment analysis and the KEGG^2^ [37] database for the pathway enrichment analysis of genes.

### Gene set enrichment analysis (GSEA)

We performed the gene set enrichment analysis as a validation method to test whether the predicted anti-COVID-19 repurposed drugs can counteract the gene expression perturbations caused by the virus. To this end we utilized the Enrichr [38] database to perform the Connectivity Map (CMAP) analysis [39]. The main concept of CMAP analysis is to compare a disease-specific gene signature with the drug-specific gene expression profiles using a comprehensive perturbation database like Connectivity Map [39, 40] that elucidate the connections between diseases, genes and drugs. We used our data set containing 524 genes as COVID-19-host signatures to evaluate the therapeutic effects of predicted drugs. To perform the CMAP analysis we submitted our genes in Enrichr database to retrieve the genes expressed up or down in cells treated with different drugs. Two data sets named CMAP-up and CMAP-down containing genes up-regulated or down-regulated respectively by various drugs were extracted. We searched for our identified repurposed drugs in CMAP data sets.

## Results

### PPI network and clustering analysis

The PPI network of 524 genes was extracted from the String database (Fig. 1). Details of the PPI network are provided in supplementary file S6. Then, clustering analysis was performed to find highly interacted modules of proteins. Seven modules of proteins were found, which are shown in Fig. 2 (More information related to these modules is provided in supplementary file S7).

### mRNA-miRNA bipartite sub-networks

**Fig. 3:**
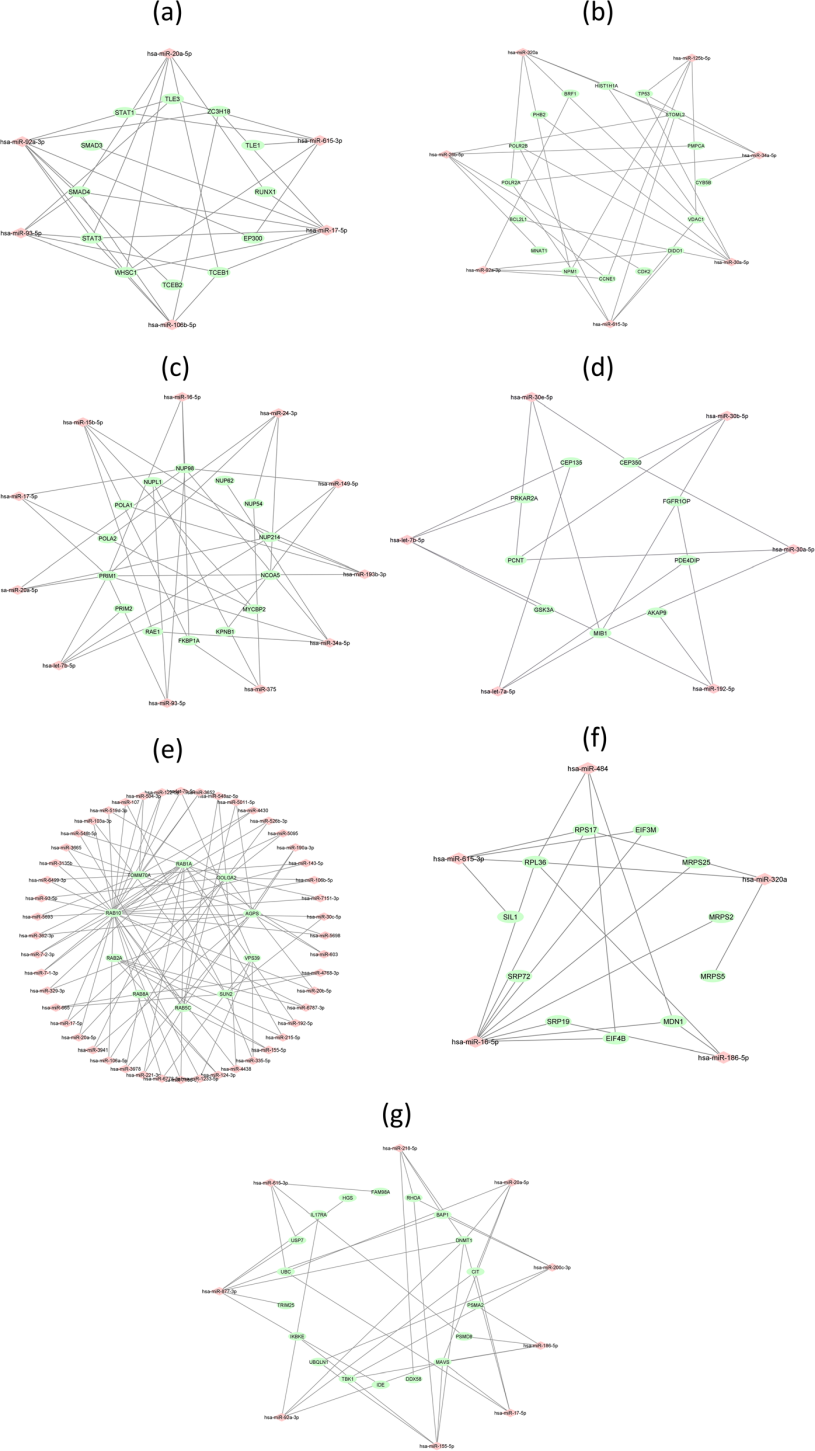
mRNA-miRNA Bipartite Sub-networks. *Note*. Green circles and red diamonds represent the genes and the miRNAs, respectively. **a** sub-network 1, **b** sub-network 2, **c** sub-network 3, **d** sub-network 4, **e** sub-network 5, **f** sub-network 6, and **g** sub-network 7.

Seven mRNA-miRNA bipartite sub-networks were reconstructed from the determined PPI modules and their targeting miRNAs. Table 1 presents the detailed properties of these networks (These networks are available as supplementary files S1 in detail). miRNAs with a higher degree in the network are more effective with a more important role in post-transcriptional gene regulation processes. Eventually, high-degree miRNAs (i.e., hub miRNAs) and the associated target genes were selected as a sub-network of the original network for further investigations so that to reduce the analysis complexity. Figure 3 depicts these seven mRNA-miRNA bipartite sub-networks. The details of these seven mRNA-miRNA bipartite sub-networks are available in supplementary file S2.

**Table 1:** The details of the mRNA-miRNA sub-networks

Cluster sub-networks	mRNA no.	miRNA no.	Interactions no.
Sub-network-1	21	532	678
Sub-network-2	23	431	557
Sub-network-3	15	409	493
Sub-network-4	14	300	364
Sub-network-5	13	385	433
Sub-network-6	14	200	234
Sub-network-7	24	360	463

*mRNA* Messenger RNA, *miRNA* microRNA

According to our previously explained hub-node selection criteria, some hub miRNAs and their target genes were considered as a sub-network for each cluster network. Sub-network 1 contains 18 nodes (6 miRNAs and 12 genes) and 34 interactions. In addition, sub-networks 2–7 include 23, 25, 15, 58, 16, and 27 nodes, as well as 32, 38, 19, 96, 22, and 39 interactions, respectively (Fig. 3 and Supplementary file S2). The list of miRNAs for sub-networks is reported in Table 2.

**Table 2:** The details of the mRNA-miRNA Sub-networks

Sub-network no.	miRNA no.	List of miRNAs
**1**	6	miR-106b-5p, miR-17-5p, miR-20a-5p, miR-615-3p. miR-92a-3p, miR-93-5p
**2**	7	miR-106b-5p, miR-17-5p, miR-20a-5p, miR-615-3p, miR-92a-3p, miR-93-5p
**3**	11	let-7b-5p, miR-149-5p, miR-15b-5p, miR-16-5p, miR-17-5p, miR-193b-3p, miR-20a-5p, miR-24-3p,miR-34a-5p, miR-375, miR-93-5p
**4**	6	let-7a-5p, let-7b-5p, miR-192-5p, miR-30a-5p, miR-30b-5p, miR-30e-5p
**5**	48	miR-93-5p, miR-7156-3p, miR-7151–3p, miR-7-2-3p, miR-7-1-3p, miR-6787-3p, miR-6778-5p, miR-665, miR-6499-3p, miR-603, miR-5698, miR-5693, miR-548 t-5p, miR-548az-5p, miR-526b-3p, miR-519d-3p, miR-5095, miR-504-3p, miR-5011-5p, miR-4768-3p, miR-4438, miR-4430, miR-3978, miR-3941, miR-3665, miR-3652, miR-362-3p, miR-335-5p, miR-329-3p, miR-3135b, miR-30c-5p, miR-221–3p, miR-215-5p, miR-20b-5p, miR-20a-5p, miR-192-5p, miR-190a-3p, miR-17-5p, miR-155-5p, miR-143-5p, miR-124-3p, miR-1233-5p, miR-122-5p, miR-107, miR-106b-5p, miR-106a-5p, miR-103a-3p, let-7b-5p
**6**	5	miR-16-5p, miR-186-5p, miR-320a, miR-484, miR-615-3p
**7**	9	miR-155-5p, miR-17-5p, miR-186-5p, miR-200c-3p, miR-20a-5p, miR-218-5p, miR-615-3p, miR-877-3p, miR-92a-3p

*mRNA* Messenger RNA, *miRNA* microRNA

Totally, 92 hub miRNAs were identified in all seven sub-networks of which 69 cases were unique miRNAs.

### Functional annotation and pathway enrichment analysis of PPI modules in SARS-CoV-2 infection

To study the underlying biological functions of genes in gene clusters, the DAVID [36, 41] was used to apply GO enrichment and pathway analysis. It should be noted that only those significant terms with *P* < 0.01 were considered in this regard. The findings of the GO enrichment analysis for each cluster of genes are provided as supplementary file S4. As reported in this file, the most significant terms for PPI-modules 1–7 were transcription from RNA polymerase II promoter, cell cycle G1/S phase transition, mitotic cell cycle phase transition, single-organism organelle organization, endomembrane system organization, translation, and negative regulation of type I interferon production, respectively.

The KEGG database was used for the pathway enrichment analysis of the genes in each PPI-module and the most significant pathway for every PPI-module, except for PPI-module_4 is reported in Table 3. There was no significant pathway for PPI-module_4. Other significant pathways for all seven PPI-modules are listed in supplementary file S5.

**Table 3:** The Significant Biological KEGG Pathways for All Seven Clusters

Cluster no.	KEGG_PATHWAY	***p***-Value
PPI_module_1	*Pathways in cancer*	1.436E-8
PPI_module_2	*Small cell lung cancer*	6.149E-5
PPI_module_3	*RNA transport*	1.072E-6
PPI_module_4	*There is no significant pathway*	–
PPI_module_5	*AMPK signaling pathway*	0.003
PPI-module_6	*Ribosome*	3.936E-4
PPI-module_7	*RIG-I-like receptor signaling pathway*	9.115E-6

*KEGG* Kyoto encyclopedia gene and genomes

### Identification of candidate drugs as a gene regulator

To identify some candidate drugs for repurposing against SARS-CoV-2 as potential therapies, the DGIdb^3^ [42] was used to consolidate the information of drug-gene interactions from multiple databases.

**Fig. 4:**
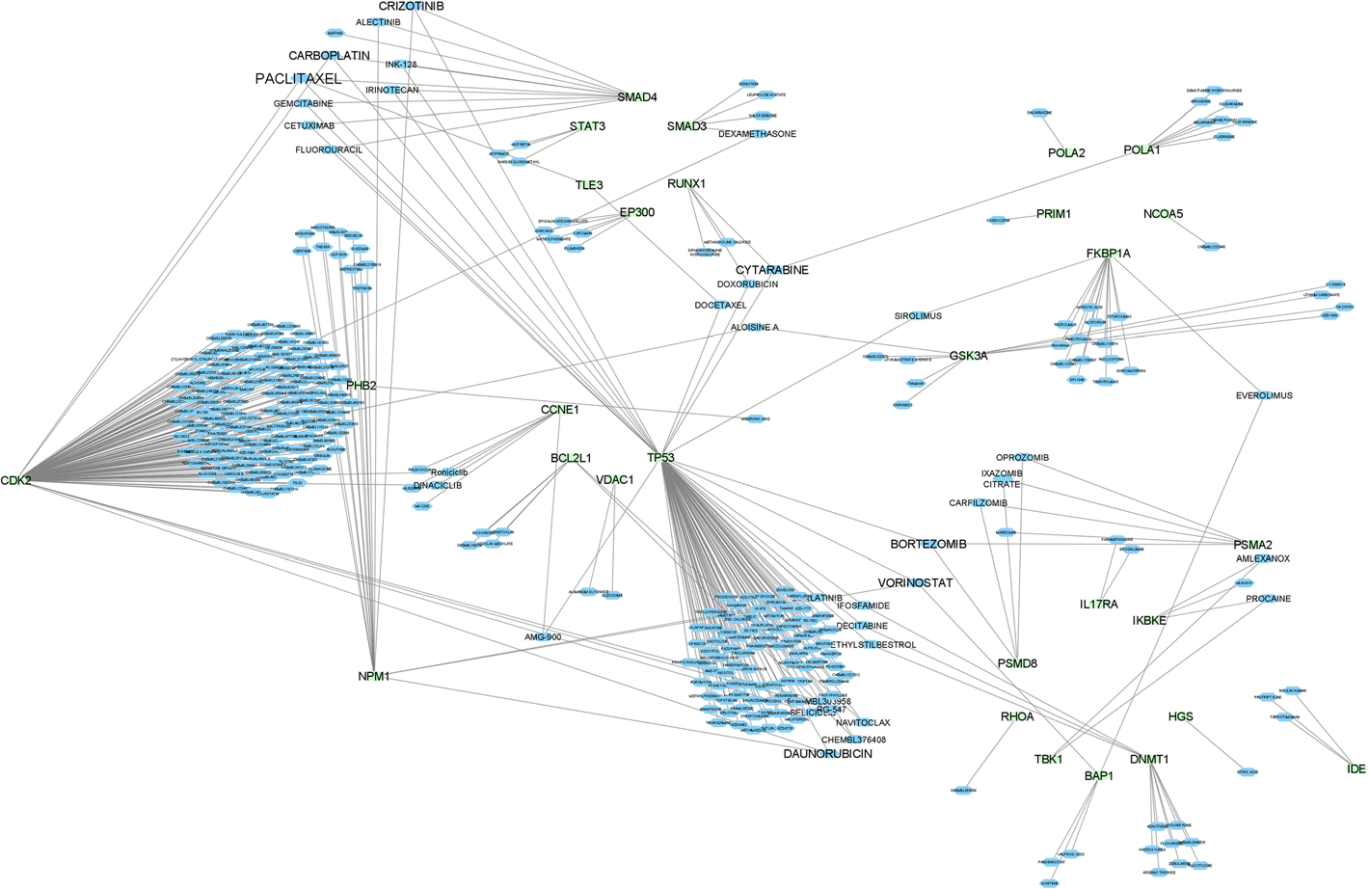
Drug-Gene Interaction Network. *Note*. The blue hexagon and green circle nodes are drugs and PPI module genes, respectively. The higher degree of the drug node represents a larger related font size. *PACLITAXEL* has four and *BORTEZOMIB*, *CARBOPLATIN*, *CRIZOTINIB*, *CYTARABINE*, *DAUNORUBICIN*, and *VORINOSTAT* have three target genes

The DGIdb was applied to identify drugs that target module genes. In this regard, first, module genes were separately imported into DGIdb, and then drug-gene interactions were obtained for module genes by limiting drugs to approved drugs. After obtaining drug-gene interactions for all modules, the entire drug-gene interactions were gartered and reconstructed as a single drug-gene network (Fig. 4).

Using this database, 28, 267, 26, 9, and 31 drugs were found for PPI_module_1, PPI_module_2, PPI_module_3, PPI_module_4, and PPI_module_7, respectively. However, no drug was found for clusters 5 and 6 PPI modules. Then, the cytoscape (Version 3.6) was utilized to visualize these data through a drug-gene network (Fig. 4). As shown in this network, some drugs have more than degree one, implying that these drugs regulate more than one gene and thus are more important in the COVID-19 gene network. According to our findings, the most remarkably identified candidate drugs for COVID-19 were *PACLITAXEL* with four interactions and *BORTEZOMIB*, *CARBOPLATIN*, *CRIZOTINIB*, *CYTARABINE*, *DAUNORUBICIN*, and *VORINOSTAT* with three interactions with the genes associated with the coronavirus infection. The above-mentioned drugs can be repurposed for treating COVID-19. More related drugs and their obtained interacted genes are available in detail in supplementary file S3.

### Gene set enrichment analysis and candidate drugs validation

In the last step to further evaluate the validation of repurposable drugs against SARS-CoV-2, we performed GSEA by querying the Enrichr CMAP database. Two data sets named CMAP-up and CMAP-down respectively containing 5667 and 5164 drugs and associated up-regulated or down-regulated genes obtained. We considered our network-based identified drugs which had contractions with 2 genes at least and searched for them in CMAP data sets. As it is shown in Table 4, a number of 11 repurposable drugs which identified in previous analysis are validated by CMAP analysis. Each row in Table 4 demonstrates the drug with related degree in obtained gene-drug network along with affected up-regulated and down-regulated genes. According to the results, PACLITAXEL, DAUNORUBICIN and VORINOSTAT that were the most potent therapeutic candidate drugs are specially validated by GSEA.

**Table 4:** The validated candidate drugs by CMAP analysis

Drug	Degree	UP	DOWN
PACLITAXEL	4	ARL4D, NUP214, LARP7, NEK9, C20ORF27, TOR1AIP1	POLA1, CCNE1, BSG, FKBP10, GTF2F2, HMOX1
DAUNORUBICIN	3	PCM1, NLRX1, C20ORF27, HMOX1	PLEKHF2, TIMM9, PRIM1, CLIP4
VORINOSTAT	3	SLC9A3R1, ATP6V1A, KDELC1, NEU1, ARL4D, ERO1LB, PDE4DIP	CCND1, H2AFY2, ZC3H7A, CDKN1B, USP13, MEPCE
DOXORUBICIN	2	FKBP10, TPSB2, ARL4D	PLEKHF2, DNAJB1
DEXAMETHASONE	2	GSK3A, BRF1, C20ORF27, NUP214, HOOK1, POR	FKBP10, NLRX1, TRIM25, LCP1, HMOX1, RALA, SLC46A3, NPTX1
IRINOTECAN	2	BRD2, NGDN	GSK3B, TIAM1
DIETHYLSTILBESTROL	2	ERO1LB, CEACAM5, ALB, INHBE, CLIP4	FOXRED2, PLOD2, POLA1, PLD3, NOL10
DECITABINE	2	ERP44,	FKBP10,
IFOSFAMIDE	2	NEK9, RAB2A, LOX,	FKBP10, RCAN3,
SIROLIMUS	2	G3BP1, DIDO1, AKAP8L, SUN2, DDAH2, CEACAM5, CEP68, SIGMAR1, NUP88, PDE4DIP, ITGB1, NOL10, POFUT1, NLRX1, FKBP10, F2RL1, ERP44, COL6A1, IFIH1, BRF1, TAPT1, IKBKB, SCARB1, DPY19L1, SMAD3, LOX, SIRT5, CHPF, NEK9, SMAD3, HMOX1, ERMP1, POFUT1, PDE4DIP, PRIM2,	CCNE1, FOXRED2, SUN2, CHEK2, POR, CHPF, RALA, GOLGA3, FKBP10, DDX58, PRKAR2B, TIMM9, CCDC86, SGTA, TRIM25, NMB, FASTKD5, ERO1LB, STC2, VPS11, HMOX1, DDX10, NOL10, RRP9, SIL1,
PROCAINE	2	G3BP1, NUP88, MASP2	SLC46A3, SLC9A3R1, PLD3

Each row demonstrates the drug with related degree in obtained gene-drug network along with affected up-regulated and down-regulated genes

## Discussion

At present, due to the emergency of finding a proper drug for the treatment of COVID-19, many studies are conducted based on drug repurposing strategy to rapidly identify an effective drug from the existing ones based on various host molecular factors [26, 43, 44]. This study analyzed the genes engaged in COVID-19 using a bioinformatics computational approach to identify candidate drugs for repurposing. To this end, the PPI network for coronavirus protein interactions was extracted from the String database. Then, for further analysis, seven clusters of proteins were specified as the complexes of proteins, which are more associated with SARS-CoV-2, followed by retrieving miRNAs associated with the identified clusters of genes. The central idea was to discover novel therapeutic candidate drugs to control gene regulation in COVID-19. Accordingly, the experimentally validated drugs were extracted from DGIDB. Recently, a similar network-based approach has been established to identify dysregulated genes and miRNAs in correlation with breast cancer [45] which demonstrates its feasibility to apply these kinds of studies for other diseases including COVID-19. Fiscon et al. [46] presented a novel network-based algorithm named SAveRUNNER^4^ regarding drug repurposing for COVID-19. In comparison with our network-based strategy that introduces a gene-drug network, they constructed a drug-disease network in which nodes are both drugs and diseases, and edges are significant associations between drugs and diseases. They applied the algorithm on 14 diseases related to the COVID-19. Focusing on known genes related to SARS with the highest genetic similarity with SARS-CoV-2, they detected 282 candidate repurposable drugs for SARS-CoV-2. In another study, Zhou et al. [26] introduced an integrative drug repurposing strategy to quantify the interactions between the human coronavirus host interactome and drug targets in the human PPI network. For building the COVID host intractome network, they assembled the host proteins of known COVID viruses such as SARS-CoV and MERS-CoV from the literature (119 COVID associated host proteins). Then, they collected drug-target interactions using multiple drug databases and mapped them to the COVID host intractome network. Finally, they identified 16 potential repurposable drugs targeting SARS-CoV-2 by applying network proximity analyses between drug targets and COVID-associated proteins.

A combination of multiple therapeutic agents including hydroxychloroquine, chloroquine, remdesivir, antiviral drugs, and common antibiotics was used for treating patients with COVID-19 [47]. Through long-time clinical uses, there is sufficient evidence of efficacy and safety regarding the application of these treatments for patients infected by the SARS-CoV-2 virus [48, 49]. However, some early studies expressed contradictory ideas in this regard. For instance, Nucsok et al. reported the potential adverse effect of hydroxychloroquine and chloroquine on cardiac function [50]. COVID-19 is a new disease and our related information is limited, and a large proportion of COVID-19 patients have underlying diseases such as cardiac injury. Thus, it is important to investigate the clinical safety of the proposed novel drugs in addition to their efficacy. Our approach in this study focused on the effectiveness of repurposed drugs and clinical safety remains unknown.

The most potent therapeutic candidate drug was *PACLITAXEL*, which is a clinical anti-cancer agent with antiviral activity [51]. Recently, Al-Motawa et al. [52] established a study applying bioinformatics tools, proteomics data, and a cell model in SARS-CoV-2 to find a suitable drug for COVID-19 based on the enrichment of arginine residues. For this purpose, the reference sequences of the SARS-CoV-2 proteome and the sequences of the human proteome were retrieved, followed by performing receptor binding domain analysis. They revealed that the anti-proliferative activity of *PACLITAXEL* leads to an increase in the concentration of methylglyoxal in cells, thus they suggested that this drug has the potential for repurposing as a candidate for the treatment of COVID-19.

The other six candidate drugs included *BORTEZOMIB*, *CARBOPLATIN*, *CRIZOTINIB*, *CYTARABINE*, *DAUNORUBICIN*, and *VORINOSTAT*, some of which were previously discovered to be efficient against COVID-19. *BORTEZOMIB* is an anti-tumor agent whitch induces apoptosis in different kinds of cancers [53]. In a study by Xing et al., *BORTEZOMIB* was reported to have reversal effects against SARS-CoV-2-induced gene expression [54]. They used 430 samples infected by MERS-CoV or SARS-CoV from different databases to extract differentially expressed genes as disease signatures for the prediction of COVID-19 candidate drugs.

Based on the findings of the current study, *VORINOSTAT* was the other striking drug, which is an anticancer histone deacetylase (HDAC). A recent study was conducted to identify repurposed drugs in SARS-CoV-2 infection [55] by analyzing dis-regulated genes in response to coronavirus infections including SARS-CoV-2. To this end, a meta-analysis approach was utilized to find common differentially expressed genes in the human hosts infected by various kinds of respiratory viruses. Accordingly, 31 up-regulated genes and 27 drugs for their regulation were found for SARS-CoV-2 cases. Among the reported drugs, *VORINOSTAT* was observed as an inhibitor of HDAC [55]. Moreover, Sinba et al. [56] reported *VORINOSTAT* as the up-regulator of angiotensin-converting enzyme 2 (ACE2), which is the human host receptor of SARS-CoV-2 in cell lines.

The next drug was *DAUNORUBICIN*, which was previously detected as an inhibitor of SARS-CoV-2 Mpro (Main protease) and reported as a potential therapeutic drug targeting COVID-19 [57]. In addition, *DAUNORUBICIN* was approved as an anti-cancer drug by the US Food and *Drug* Administration regulating SARS-CoV-2 interactors (The ABCC1 gene) and having the potential for investigation as a repurposed drug against SARS-CoV-2 infection [58, 59].

Based on the literature review, no study was found to report *CARBOPLATIN*, *CRIZOTINIB*, and *CYTARABINE*, which were introduced in the current study. Therefore, these novel potential drugs should be investigated as a therapy for COVID-19.

This study had some limitations. The study could not investigate the validation of these drugs in vitro and in vivo due to the computational nature of our study method and the restriction of experimental resources. Nevertheless, regarding the imperative need to reach treatment for COVID-19, these identified candidate drugs are required to be validated in experimental studies. Moreover, as mentioned above, our approaches only considered the efficacy aspect of drugs, and thus urgent clinical trials are needed to provide safety data on repurposed drugs for the treatment of COVID-19 patients.

In conclusion, as a strategy for predicting repurposable candidate drugs against COVID-19, the protein-protein interaction network-based method was used to find genes and related miRNAs highly correlated with coronavirus. These computational methods provide efficacious and rapid data for therapeutic purposes. However, further experimental analysis and testing such as clinical applicability, toxicity, and experimental validations are required to reach a more accurate and improved treatment. Our proposed complexes of proteins and associated miRNAs, along with the discovered candidate drugs might be a starting point for further analysis in this urgency of COVID-19 pandemic.

## Supplementary Information

**Additional file 1.**

**Additional file 2.**

**Additional file 3.**

**Additional file 4.**

**Additional file 5.**

**Additional file 6.**

**Additional file 7.**

## Data Availability

The data used in this project is available at (https://github.com/habibmoti/COVID-19). All data, raw and processed, is readily available from the corresponding author on request.

## Footnotes

1 Database for Annotation Visualization and Integrated Discovery

2 Kyoto Encyclopedia Gene and Genomes

3 Drug Gene Interaction database

4 Searching off-lAbel dRUg aNd NEtwoRk
